# Daidzein induces neuritogenesis in DRG neuronal cultures

**DOI:** 10.1186/1423-0127-19-80

**Published:** 2012-08-29

**Authors:** Shih-Hung Yang, Chih-Chen Liao, Ying Chen, Jhih-Pu Syu, Chung-Jiuan Jeng, Seu-Mei Wang

**Affiliations:** 1 Division of Neurosurgery, Department of Surgery, National Taiwan University Hospital, Taipei, Taiwan; 2 Department of Anatomy and Cell Biology, College of Medicine, National Taiwan University, No. 1, Section 1, Jen-Ai Road, Taipei 10051, Taiwan; 3 Department of Biology and Anatomy, National Defense Medical Center, Taipei, Taiwan; 4 Institute of Anatomy and Cell Biology, School of Medicine, National Yang-Ming University, No. 155, Section 2, Li-Non Street, Taipei 12212, Taiwan

**Keywords:** Daidzein, DRG neuronal culture, Neurite outgrowth, Signaling mechanism

## Abstract

**Absract:**

**Background**

Daidzein, a phytoestrogen found in isoflavone, is known to exert neurotrophic and neuroprotective effects on the nervous system. Using primary rat dorsal root ganglion (DRG) neuronal cultures, we have examined the potential neurite outgrowth effect of daidzein.

**Methods**

Dissociated dorsal root ganglia (DRG) cultures were used to study the signaling mechanism of daidzein-induced neuritogenesis by immunocytochemistry and Western blotting.

**Results**

In response to daidzein treatment, DRG neurons showed a significant increase in total neurite length and in tip number per neuron. The neuritogenic effect of daidzein was significantly hampered by specific blockers for Src, protein kinase C delta (PKCδ) and mitogen-activated protein kinase/extracellular signal-regulated kinase kinases (MEK/ERK), but not by those for estrogen receptor (ER). Moreover, daidzein induced phosphorylation of Src, PKCδ and ERK. The activation of PKCδ by daidzein was attenuated in the presence of a Src kinase inhibitor, and that of ERK by daidzein was diminished in the presence of either a Src or PKCδ inhibitor.

**Conclusion**

Daidzein may stimulate neurite outgrowth of DRG neurons depending on Src kinase, PKCδ and ERK signaling pathway.

## Background

Daidzein, found mainly in soy food products and herbs like red clover [1,2], is one of the most studied and most potent phytoestrogens. Phytoestrogens are estrogenic compounds of plant origin, and have structures and functions similar to the mammalian endogenous hormone estrogen [3]. Both estrogen and phytoestrogens can bind to intracellular estrogen receptors (ERs) to trigger downstream signal transduction pathways and achieve various biological functions [4]. Phytoestrogen acts mainly as an ER agonist. It may also function as an antagonist, by inhibition of aromatase activity in breast cancer cells, and blockage of estrogen uptake by uterine cells [5]. This mixed ER agonist/antagonist property probably explains the potential benefit of phytoestrogen in breast cancer prevention [6-8].

In response to ligand binding, ERs can signal through both genomic (classical) and non-genomic (non-classical) pathways [9,10]. In the genomic pathway, upon binding to estrogen, ERs dimerize and interact with the estrogen responsive element (ERE) in the regulatory regions of estrogen responsive genes, thereby regulating the transcription of E2-sensitive genes, e.g. c-fos, TGF-α, and angiotensinogen [9]. The non-genomic pathway involves the activation of other signal transduction pathways that lead to rapid and diverse physiological responses, including calcium and potassium influxes through cell membrane, and activation of second messenger systems such as cAMP/PKA, MAPK, PI3K/Akt, and G protein [10,11]. The precise mechanisms of non-genomic effects of estrogen are not clear and have been suggested to be mediated by membrane-associated ERα, ERβ, or the orphan G-protein-coupled receptor 30 (GPR30) [12,13]. Activation of non-nuclear ERα, for example, can stimulate endothelial cell proliferation via G protein, Src, and eNOS activation [14]. In cultured osteoblastic cells, daidzein has been suggested to activate a non-classical membrane ER-β pathway that involves phospholipase C-β2 (PLC-β2)/PKC and PI3K/cSrc [15].

Emerging evidence, however, indicates that for some of the phytoestrogen effects, ER activation may not be required. For instance, genistein, a rich phytoestrogen in soybeans, was shown to exert growth inhibitory effects in ER-negative breast cancer cells [16-18]. Compared to 17-β estradiol, the most biologically active estrogen in mammals, daidzein has a significantly lower affinity for both ER-α and ER-β [19]. Moreover, daidzein could induce anti anti-proliferative effects in both ER-positive and ER-negative pancreatic cells [20]. Together these observations raise the possibility that daidzein may also exert its pharmacological effect via an ER-independent signaling pathway.

Daidzein is known to exert significant neuronal protection and neuritogenic effects for a variety of cultured neuronal cells, e.g. hippocampal neurons, cortical neurons, dorsal root ganglion (DRG) neurons, and PC12 cells [21-24]. In hippocampal neuron, the neuritogenic mechanism involves ERβ-PKCα-GAP43 signaling. To further understand the diversity of the intracellular signaling mechanisms of daidzein, in the current study we focused on daidzein-induced neurite outgrowth in cultured DRG neurons. DRG culture is a well-characterized system for investigating the mechanism of neuritogenesis [25-27], and for screening neuroprotective drugs for peripheral neuropathies [28]. Studies using DRG cultures have shed light on the pathogenic mechanisms of peripheral nervous system diseases and the regeneration of spinal cord injury [29-31]. Here we showed that in cultured DRG neurons daidzein induced notable neuritogenesis via an ER-independent signaling pathway. In addition, we presented several lines of evidence suggesting that daidzein-induced neurite outgrowth in DRG neurons may be primarily mediated by the Src kinase, PKCδ and ERK signaling pathway.

## Methods

### Drugs

Daidzein was purchased from the Pharmaceutical Industry Technology and Development Center (New Taipei City, Taiwan). Nerve growth factor (NGF) was purchased from R&D Systems (Minneapolis, MN, USA). Dimethyl sulfoxide (DMSO), antibodies for neurofilament light chain (NF-L) were purchased from Sigma Chemical Co. (St. Louis, MO, USA). L-15 Leibovitz medium was purchased from Gibco (Grand Island, NY, USA). ER antagonists ICI182780, tamoxifen and G-protein coupled receptor 30 (GPR-30) antagonist G15 were obtained from TOCRIS (TOCRIS Cookson Inc., Bristol, UK). Src kinase inhibitor PP2, MEK inhibitor U0126, PKC inhibitor staurosporin, and PKCδ inhibitor rottlerin were purchased from Biomol Research Laboratory Inc. (Plymouth meeting, PA, USA).

### Animals

Postnatal day 2 Wistar rat pups were purchased from the Facility for Animal Research of the National Taiwan University. All procedures were in accordance with the *Guidelines for the Care and Use of Mammals in Neuroscience and Behavioral Research* (National Research Council 2003) and approved by the Institutional Animal Care and Use Committee (IACUC) of National Taiwan University, College of Medicine.

### Cell culture

DRG cultures were prepared as described previously [25]. Briefly, P2 rat pups were put on ice and then decapitated to harvest DRG. DRG were then dissected out under microscope and dissociated with 0.25% trypsin and 0.05% collagenase (Sigma) in HBSS solution, for 30 min at 37°C. These ganglia were then dispersed by mechanically trituration with glass pipettes. The pellet from low-speed centrifugation was re-suspended in phenol-red free L-15 Leibovitz media, supplemented with 1.2 g/L of NaHCO_3_, 5% fetal bovine serum, 100 IU/mL of penicillin, and streptomycin (Gibco). Cells were plated on collagen-coated coverslips for immunocytochemistry, and on 35 mm uncoated culture dishes for protein quantification by Western blot. The medium was changed to serum free L-15 for day in vitro (DIV) 2 cultured DRG cells. Cultures were maintained at 37°C in an atmosphere of 95% air and 5% CO_2_.

### Cell survival assay

The MTT assay, a colorimetric assay for measuring the activity of mitochondrial enzymes, was used to examine whether cell viability was affected by treatmen [32]. In each well of 24-well culture plates, 2 × 10^4^ cells were plated and were treated with 0.1% DMSO, different concentration of daidzein or different kinase inhibitors for 24 h. After treatments, cells were washed with phosphate-buffered saline (PBS; 137 mM NaCl, 2.7 mM KCl, 1.5 mM KH_2_PO_4_, 8 mM Na_2_HPO_4_, pH 7.4), and incubated in 0.5 mg/ml of 3-[4, 5-dimethylthiazol-2-yl]-2, 5-diphenyltetrazolium bromide (MTT) solution for 4 h to allow the conversion of MTT into the purple formazan product by mitochondrial dehydrogenases. The reaction medium was then removed and the cells were lysed with DMSO for 5 min. The absorbance was read at 590 nm with a spectrophotometer (Beckman Coulter Inc., Fullerton, CA).

### Drug treatment

DIV 3 cultured DRG cells received either daidzein at a concentration of 10 μM, 30 μM, 50 μM, or 100 μM, vehicle solution DMSO (final concentration of 0.1%), or NGF of 100 ng/mL, in order to study the effect of daidzein on neurite outgrowth.

For inhibitor assay, one of following inhibitors was reacted 30 min before the addition of daidzein: estrogen receptor antagonists ICI182780 at 1 μM and tamoxifen at 10 μM; GPR-30 inhibitor G15 at 100 nM; Src kinase inhibitor PP2 at 10 μM; PKC inhibitor staurosporin at 100 nM; PKC α/β inhibitor Gö6976 at 1 μM; PKCϵ inhibitor ϵV1-2 at 2 μM; PKCδ inhibitor rottlerin at 2 μM; MEK inhibitor U0126 at 10 μM.

### Immunocytochemistry

After 24 h of DMSO or daidzein treatment, DRG neurons on cover glasses were fixed for 10 min with 10% formalin in PBS. After washed with PBS, cells were then permeabilized and blocked with 0.15% Triton X-100 and 5% non-fat milk in PBS for 1 h. DRG neurons were then incubated in mouse anti-NF-L antibody overnight at 4°C. After PBS wash, cells were incubated in biotin-conjugated goat anti-mouse IgG (Vector, Burlingame, CA, USA) at 1:50 dilution for 1 h at room temperature, washed with PBS, then reacted with avidin-biotinylated enzyme complex (Vector) for one hour at room temperature. Following PBS wash, staining was done with peroxidase-chromogen reaction (SG substrate kit, Vector), which was stopped by Tris-buffered saline (TBS: 50 mM Tris-Base, 150 mM NaCl, pH 8.2). Coverslips were then dehydrated by ethanol and xylene, and mounted with Permount (Fisher Scientific, NH, USA). Images were taken on a light microscope, equipped with a Nikon DIX digital camera (Nikon, Tokyo, Japan).

### Western blotting

After various treatment, the cultured DRG neurons were homogenized in ice-cold lysis buffer solution (10 mM EGTA, 2 mM MgCl_2_, 0.15% Triton X-100, 60 mM PIPES, 25 mM HEPES, pH 6.9, containing 1 μM phenylmethylsulfonyl fluoride, 1 μM NaF, 10 μg/ml of leupeptin and 1 μg/ml pepstatin) and sonicated. A 3-fold volume of 4X reducing SDS sample buffer was added to each lysate and boiled at 95°C for 5 min. Fifty microgram of protein from each sample (protein concentration determined by Bio-Rad protein Kit, Bio-Rad Lab, CA, USA) were separated by 10% polyacrylamide-SDS gel electrophoresis, electrotransferred to nitrocellulose membrane (Schleicher and Schuell, Keene, NH, USA), blocked by TBS containing 5% non-fat milk and 0.1% Tween-20, and then incubated overnight at 4°C with the following primary antibodies: rabbit anti-pTyr527-Src (Cell Signaling) at 1: 500 dilution; rabbit anti-pThr505 PKCδ (Epitomics, Burlingame, CA, USA) at 1:500 dilution and rabbit anti-PKCδ (Santa Cruz, Santa Cruz, CA, U.S.A.) at 1:500 dilution; mouse anti-pThr and anti-pTyr ERK (Sigma) at 1:1000 dilution and rabbit anti-ERK1/2 (Santa Cruz) at 1:500 dilution; mouse anti-cSrc (Millipore, Billerica, MA, USA) at 1:300 dilution. Following washes with TBS containing 0.1% Tween-20, alkaline phosphatase conjugated secondary antibodies at 1:7500 dilution (Promega, Madison, WI, USA) were added for an hour at room temperature, and the bound antibodies visualized using enzyme-substrate reaction (substrate: 3.3 mg/ml nitro blue tetrazolium and 1.65 mg/ml 5-bromo-4-chloro-3-indolyl phosphate in 100 mM NaCl, 5 mM MgCl_2_, 100 mM Tris-base, pH 9.5).

### Quantification

Immunostained neurons were photographed at 20× or 40× (for morphological demonstration) magnification, and the images transformed into 256 gray scale images. The fields were chosen to locate individual neuron with discernible neurites from nearby neurons. Generally, an average of 2–4 neurons could be seen in a micrograph taken at 20× magnification. The total neurite length was then measured from the somata using a PC-based image analyzer software Image Pro 3.0 Plus (Media Cybernetics, Silver Spring, MD, USA). The signal intensity of bands stained on immunoblot was quantified with Gel pro 3.1 (Media Cybernetics). Student’s *t*-test was used for evaluating statistical differences between the means of different groups, with p value of less than 0.05 considered significant.

## Results

### Daidzein enhances neuritogenesis in cultured DRG neurons

**Figure 1:**
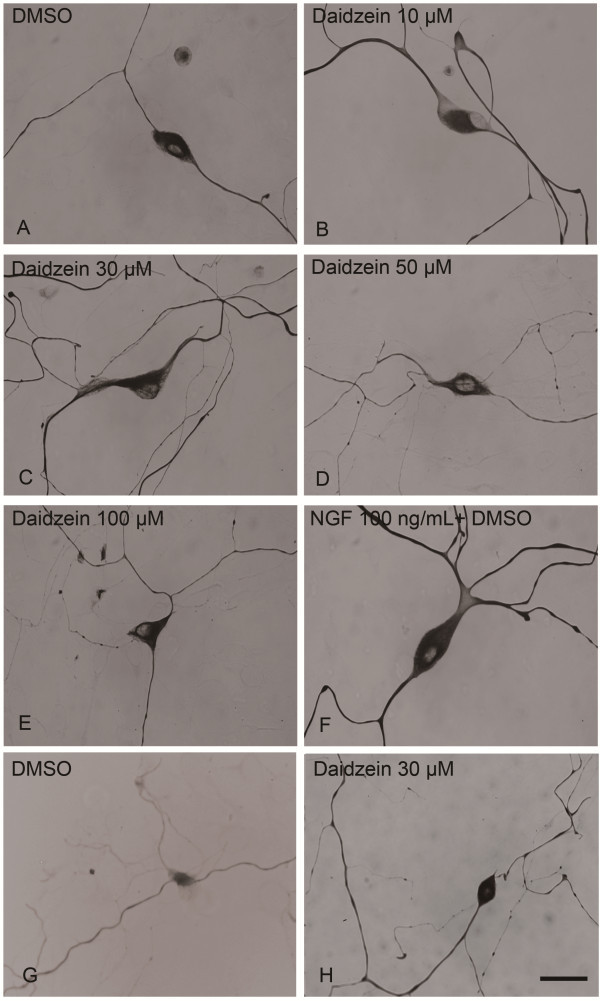
**Induction of neurite outgrowth of cultured rat DRG neurons by daidzein and NGF. **DRG neuronal cultures were treated for 24 h with (**A**) DMSO, (**B-H**) various concentration of daidzein, or (**F**) NGF (100 ng/ml), then fixed and immunostained for NF-L. A-F, large neurons. G-H, small neurons. Scale Bar = 30 μm.

**Figure 2:**
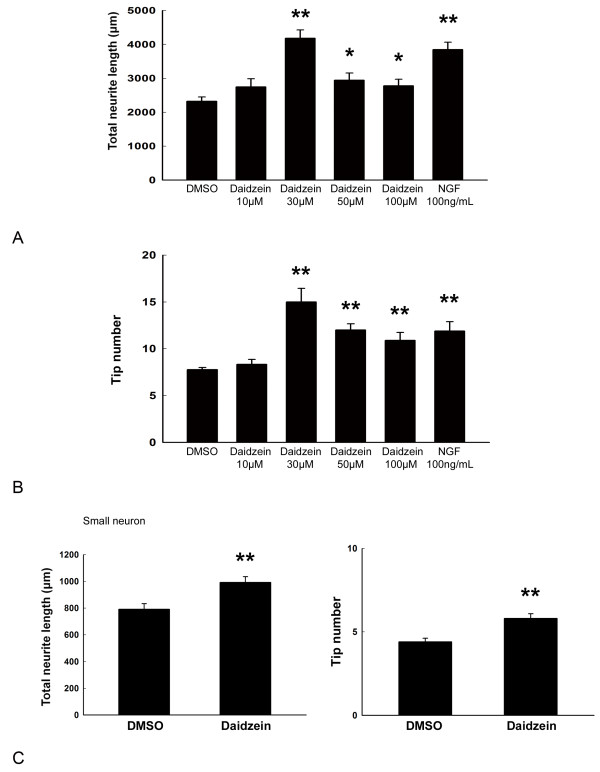
**Quantitative analysis of total neurite length and tip number of neurite branches per neuron following various treatments with DMSO, daidzein, or NGF. **Significant neurite lengthening and branching were observed in both large (**A** and **B**) and small DRG neurons (**C**) treated with 30 μM daidzein or 100 nM NGF. Three independent experiments were performed. Ten neurons were chosen from each group in one representative experiment for analysis. *, p < 0.05 vs DMSO controls. **, p ≤ 0.01 vs DMSO controls.

**Figure 3:**
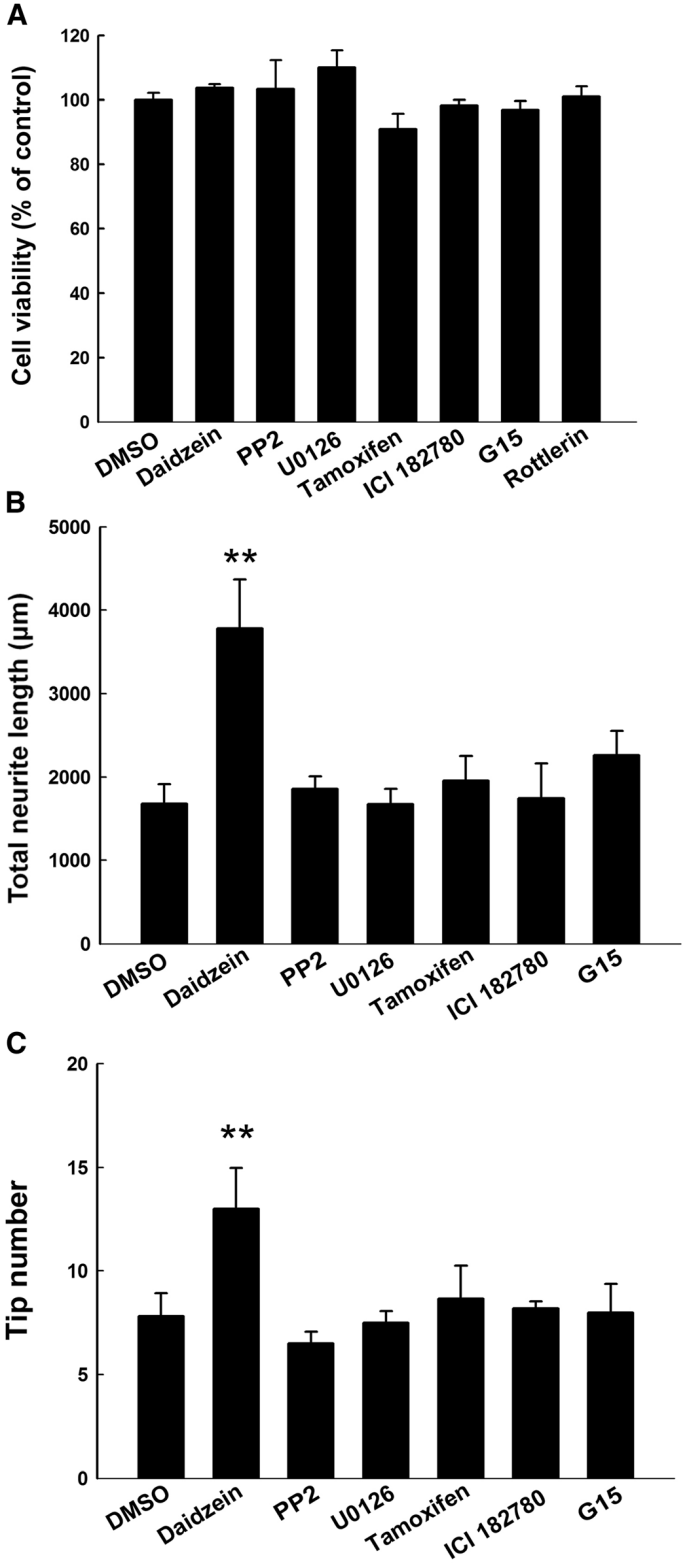
**Effect of different inhibitors on cell viability and neuritogenesis. **DRG neurons were treated with 0.1% DMSO, 30 μM diadzein, 10 μM PP2, 10 μM U0126, 10 μM tamoxifen, 1 μM ICI182780 or 100 nM G15 for 24 h. They were then assayed by the MTT test (**A**), or immunostained for NF-L and analyzed for total neurite length (**B**) and tip numbers (**C**).

We first studied the effect of daidzein on neurite outgrowth in primary rat DRG neuronal cultures. DRG neurons were classified into large (diameter ≥ 40 μm) and small (diameter ≤ 40 μm) according to the criteria described by [Gavazzi 33]. Based on cell diameter, small-sized unmyelinated neurons that are responsible for pain sensation, and large-sized myelinated that are for proprioception. DIV 3 neurons were incubated with different concentrations of daidzein, DMSO (negative control), or NGF (positive control) for 24 h. Compared to DMSO-treated control cultures, daidzen treatment significantly enhanced neurite extension and branching of large DRG neurons (Figures 1,2A-B). Quantitative analysis of total neurite length and tip numbers per neuron indicated that the minimal effective concentration of daidzein in promoting neurite outgrowth was 30 μM (length, 4180 ± 246 μm; tip number, 15.0 ± 1.4; DMSO, 2323 ± 128 μm; tip number, 7.8 ± 0.2; p ≤ 0.01, n = 10), which had an effect similar to that of 100 ng/ml NGF (Figure 2) and was thus used for further study of the compound. The effect of daidzein-induced neuritogenesis was observed similarly in both small-sized and large-sized DRG neurons (Figures 1G-H, 2C). Furthermore, to examine whether daidzen was toxic to DRG neurons, double staining of cells with DAPI and PI was performed to identify apoptotic and necrotic cells, respectively [34]. No apparent cell death was observed at 30 μM by MTT assay (Figure 3A). 

### Src and ERK inhibitors block neuritogenic effect of daidzein

**Figure 4:**
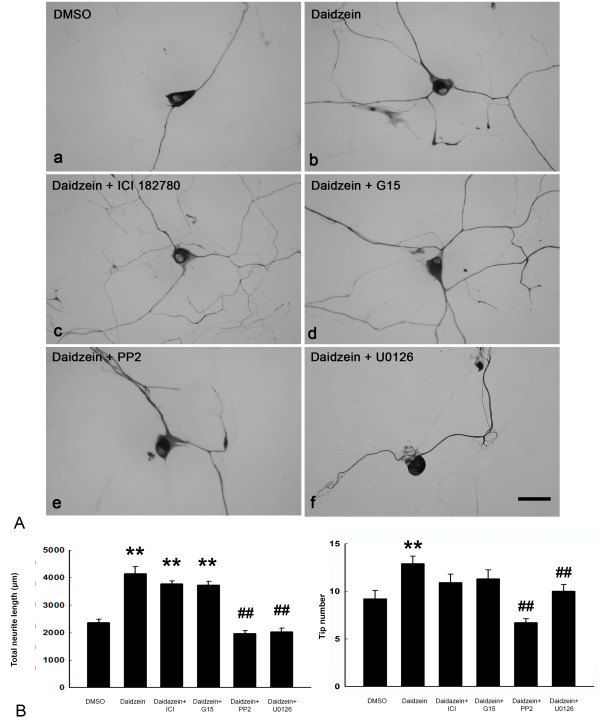
**Blockage of daidzein-induced neuritogensis by kinase inhibitors of Src and MEK, but not by ER antagonists. **(**A**) DRG neuronal cultures were treated for 24 h with (**a**) DMSO, or (**b**) 30 μM daidzein. Inhibitor assay was conducted by pretreatment of DRG neuronal cultures for 30 min with (**c**) ER antagonist ICI182780 at 1 μM, (**d**) GPR-30 antagonist G15 at 100 nM, (**e**) Src kinase inhibitor PP2 at 10 μM, or (**f**) MEK inhibitor U0126 at 10 μM, followed by 30 μM daidzein for 24 h. The neurons were fixed and immunostained for NF-L. Scale Bar = 30 μm. (**B**) Analysis of total neurite length and tip number revealed that PP2 and U0126 significantly blocked the neuritogenesis induced by daidzein. **, p ≤ 0.01 vs DMSO group; ##, p ≤ 0.01 vs daidzein group. N = 10.

To investigate the signaling mechanism underlying the neuritogenic effect of daidzein, various inhibitors were applied to DRG neuronal cultures before and during daidzein treatment. Daidzein is structurally similar to estrogen and can activate ER in other cell culture systems [15,22]. Thus, we first examined this possibility. Treatment alone with inhibitors for ER-α/β, GPR-30, Src, or MEK had no effect on total neurite length and tip numbers (Figure 3B, C). Neither the ER-α/β inhibitor ICI 182780 (length, 3778 ± 101 μm, tip number, 10.9 ± 0.9 μm; daidzein, length, 4145.8 ± 255.5 μm, tip number, 12.9 ± 0.8; DMSO, length, 2363.2 ± 128.5 μm, tip number, 9.3 ± 0.9, n = 10) nor the GPR-30 antagonist G15 (length, 3721 ± 141 μm; tip number, 11.3 ± 0.9, n = 6) blocked the daidzein-induced neurite lengthening or branching in our cultured DRG neurons (Figure 4). Another ER antagonist tamoxifen also did not inhibit daidzein-induced neuritogenesis (length, 2256.6 ± 580 μm, n = 10; DMSO, 2629.4 ± 436 μm; daidzein, 3632.7 ± 560 μm). On the other hand, both the Src inhibitor PP2 (length, 1971 ± 101 μm, tip number, 6.7 ± 0.4, n = 10, p ≤ 0.01 daidzein, length 4145.8 ± 255.5 µm, tip number 12.9 ± 0.8 control, length 2363.2 ± 128.5 µm, tip number, 9.3 ± 0.9), and the MEK inhibitor U0126 (length, 2031 ± 126 tip number, 10.0 ± 0.7, n = 10, p ≤ 0.01) significantly reduced daidzein-induced neuritogenesis (Figure 4). Treatment with PP2 or U0126 alone had no effect on cell survival, total neurite length, or tip numbers (Figure 3B, C). 

### Blocking of the neuritogenic effect of daidzein by PKCδ inhibitor

To examine whether PKC involved in the daidzein-induced neuritogenesis, we treated DRG neurons with a pan-PKC activator PMA in the presence of the src kinase inhibitor PP2. The result showed that activation of PKC by PMA could reverse the PP2-induced decrease in total neurite length (PMA + PP2 + daidzein, length, 1999 ± 85 μm; DMSO, 1768 ± 77 μm; PP2 + daidzein, 1401 ± 72 μm, p ≤ 0.05, n = 5).

**Figure 5:**
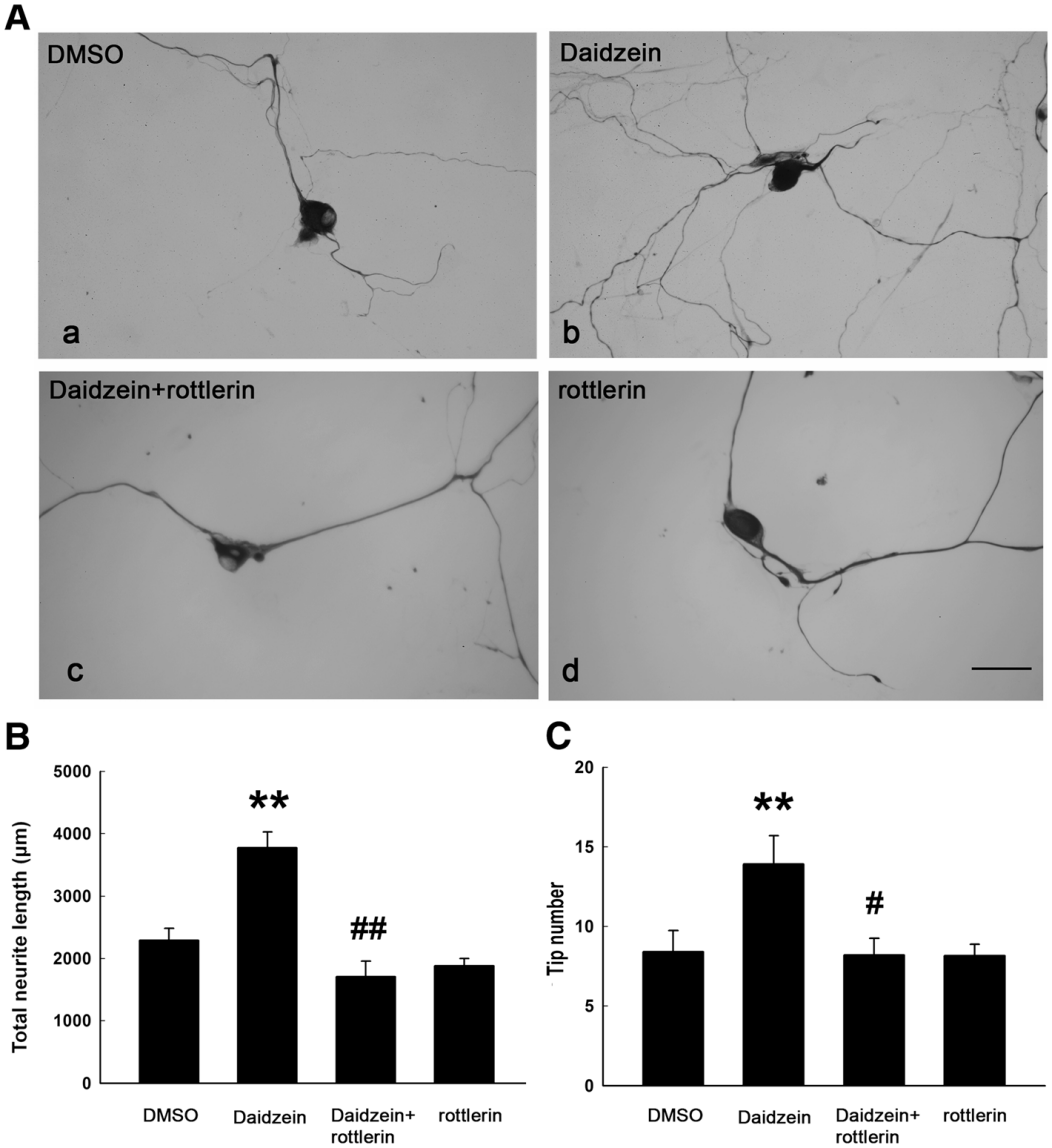
**Blockage of daidzein-induced neuritogenesis by kinase inhibitors of PKCδ. **(**A**) DRG neuronal cultures were treated for 24 h with (**a**) DMSO, (**b**) 30 μM daidzein or (**d**) 2 μM PKCδ inhibitor rotterlin. Inhibitor assay was conducted by pretreatment of DRG neuronal cultures for 30 min with (**c**) 2 μM rottlerin, followed by 30 μM daidzein for 24 h. The neurons were fixed and immunostained for NF-L. Scale Bar = 30 μm. Analysis of total neurite length (**B**) and tip number (**C**) revealed that rottlerin significantly blocked the neuritogenesis induced by daidzein. **, p ≤ 0.01 vs DMSO; #, p ≤ 0.05 vs daidzein, ##, p ≤ 0.01 vs daidzein. N = 10.

To further investigate the signaling mechanism involved in the neuritogenic effect of daidzein, various PKC inhibitors were applied to DRG neuronal cultures. Neither PKCα inhibitor Gő6976 (length and tip number, 3853 ± 673 µm and 17.4 ± 0.6; daidzein, 3382 ± 340 µm and 18.0 ± 1.0, DMSO, 1982 ± 512 µm and 10.8 ± 0.7, n = 10) nor PKCϵ inhibitor ϵV1-2 (length and tip number, 3424 ± 482 µm and 16.1 ± 1.0, n = 10) had any effect on the daidzein-induced neuritogenesis. Only the PKCδ inhibitor rottlerin (length, 1705 ± 247 μm, n = 10, p ≤ 0.01) significantly block daidzein’s neuritogenic effect (length, daidzein 3770 ± 252 μm; DMSO; 2291 ± 193 μm, n = 10) (Figure 5). Treatment with Gő6976, ϵV1-2 (data not shown) or rottlerin (Figure 5) did not affect total neurite length and tip numbers.

### Daidzein treatment activates Src, PKCδ, and ERK

**Figure 6:**
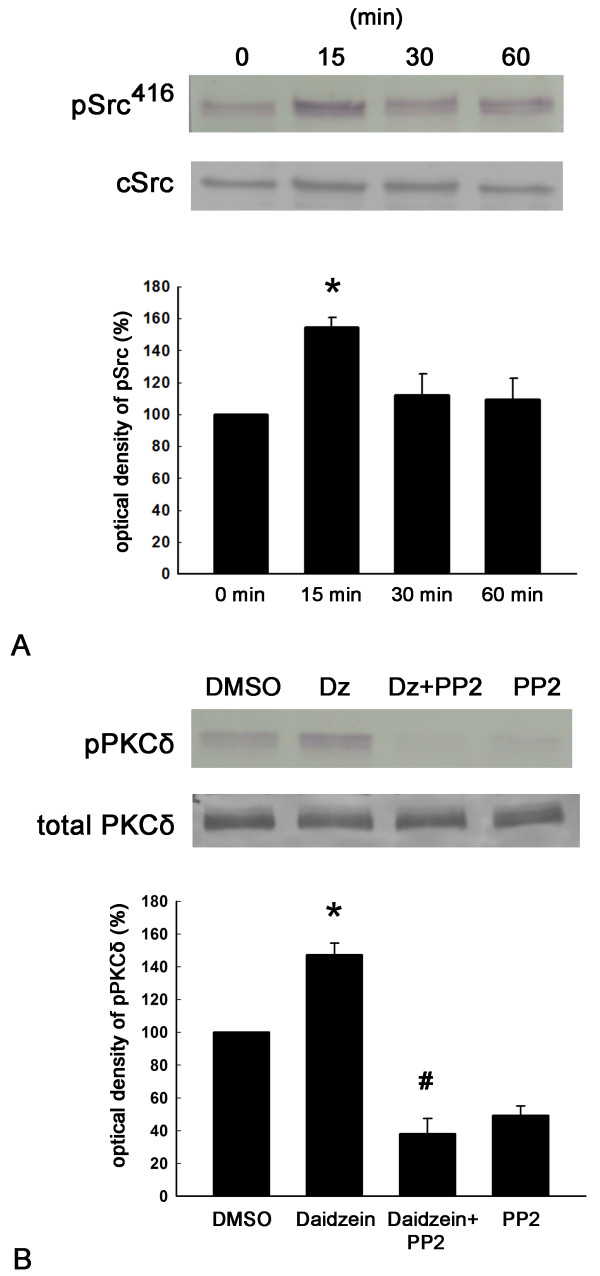
**Activation of Src and PKCδ by daidzein treatment and suppression of the PKCδ activation by the specific Src inhibitor PP2. **(**A**) Phosphorylation of Src following daidzein treatment was analyzed by Western blot analysis. DRG neuronal cultures were treated with 30 μM daidzein for 0, 15, 30, or 60 min, then the cell homogenate was analyzed for phosphorylated Src (pSrc416) and total Src. Upper panel showed a representative blot from one experiment. Lower panel showed optical densities of the densitometric scans of the pSrc416 bands. Basal levels of phosphorylation in non-stimulated cells (DMSO) were taken as 100% for each individual treatment. *, p < 0.05 vs 0 min, n = 4. (**B**) Phosphorylation of PKCδ was analyzed by Western blot analysis. DRG neuronal cultures were given DMSO, 30 μM daidzein (Dz) for 30 min, 10 μM the Src inhibitor PP2 for 30 min followed by 30 μM daidzein for 30 min (Dz + PP2), or 10 μM PP2 for 60 min (PP2). The cell homogenate was analyzed for phosphorylated and total PKCδ. Upper panel showed a representative blot from one experiment. Lower panel showed optical densities of the densitometric scans of the PKCδ bands. PP2 treatment reduced both basal and. daidzein-induced PKCδ phosphorylation. *, p < 0.05 vs DMSO; #, p < 0.05 vs daidzein. n = 4.

**Figure 7:**
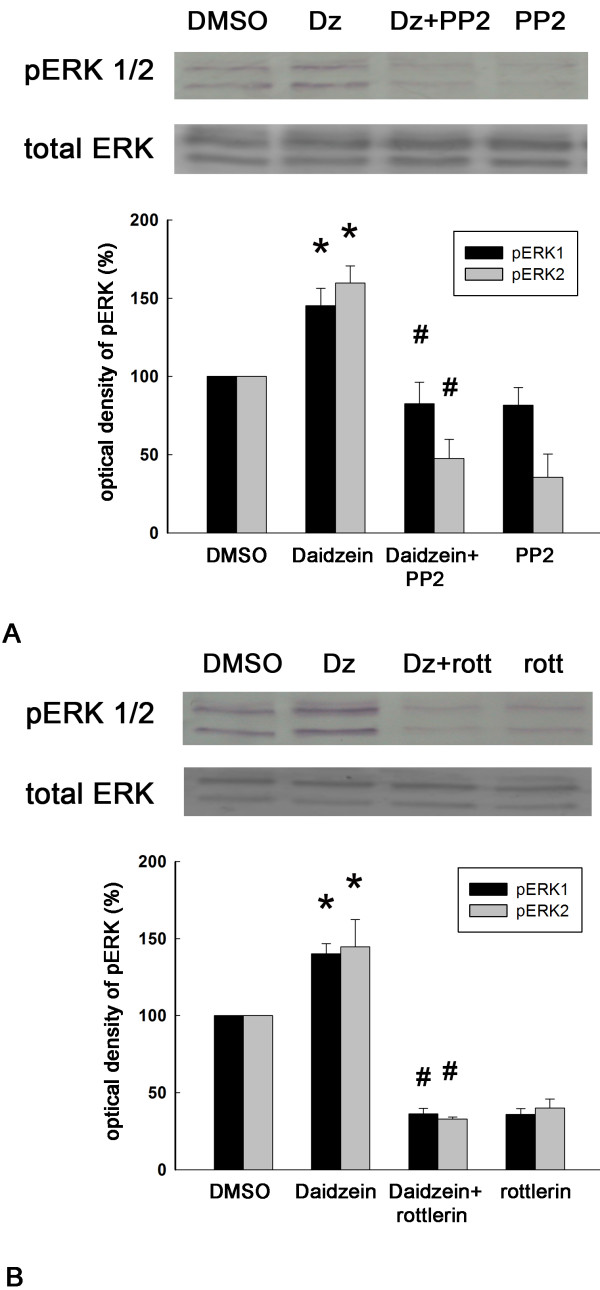
**Activation of ERK by daidzein treatment, and suppression of the ERK activation by both Src and PKCδ inhibitors. **Upper panel showed a representative blot from one experiment. Lower panel showed optical densities of the densitometric scans of the pERK bands. (**A**) DRG neuronal cultures were treated with DMSO, 30 μM daidzein (Dz) for 30 min, 10 μM the Src inhibitor PP2 for 30 min followed by 30 μM daidzein (Dz + PP2) for 30 min, or 10 μM PP2 for 60 min (PP2). The cell homogenate was analyzed for phosphorylated pERK and total ERK. *, p < 0.05 vs DMSO; #, p < 0.05 vs daidzein. n = 4. (**B**) DRG neuronal cultures were treated with DMSO, 30 μM daidzein (Dz) for 30 min, 2 μM the PKCδ inhibitor rottlerin for 30 min followed by 30 μM daidzein for 30 min (Dz + rott), or 2 μM rottlerin for 60 min (rott). The cell homogenate was analyzed for phosphorylated pERK and total ERK. *, p < 0.05 vs DMSO group; #, p < 0.05 vs daidzein group. n = 4.

**Figure 8:**
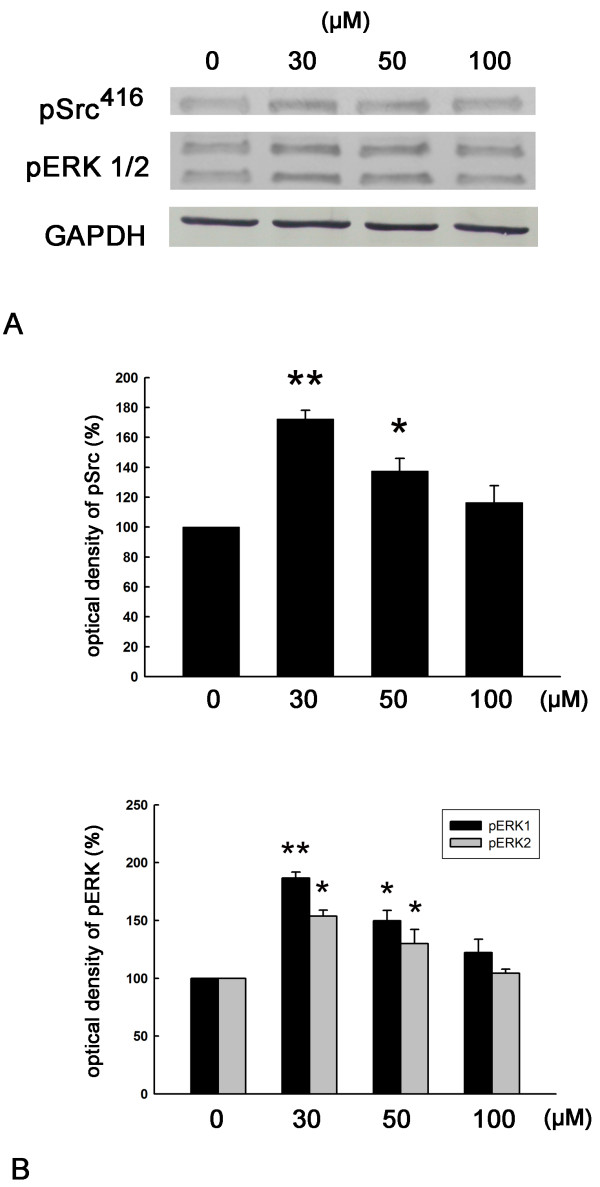
**Effect of different concentrations of daidzein on activation of Src kinase and ERK. **DRG neurons were treated with 0, 30, 50 or 100 μM daidzein for 30 min, Total cell lysates were analyzed for pSrc (**A**) and pERK (**B**). GAPDH is loading control. *, p ≤ 0.05, **, p ≤ 0.01 vs 0-min group. n = 3.

We next examined whether daidzein could activate the Src, PKCδ, or the ERK signaling pathway. By using Western blotting with antibodies directed against phosphotyrosine-416 of active Src (pSrc416), we found that the pSrc416 level was significantly increased by daidzein treatment (Figure 6A). Interestingly, pPKCδ levels were also up-regulated in the presence of daidzein treatment, and the Src kinase inhibitor PP2 significantly inhibited this activation of pPKCδ by daidzein (Figure 6B). Moreover, ERK1 and ERK2 were activated by daidzein, and this effect was suppressed by both the Src kinase inhibitor PP2 and the PKCδ inhibitor rottlerin (Figure 7). Interestingly, a dose-dependent study of daidzein showed that a maximal activation of Src kinase and ERK was found at 30 μM daidzein (Figure 8). This data correlates well with the previous observation that the optimal concentration of daidzein to achieve effective neurotigenic activity is 30 μM.

## Discussion

Previously, in cultured osteoblastic cells, daidzein was shown to bind to cell membrane ER-β to activate the phospholipase C β2 (PLC-β2)/PKC and PI3K/cSrc pathways, leading to the expression of several groups of genes for cell differentiation, proliferation, and migration [15]. Similarly, in macrophage, daidzein inhibited the activation of STAT-1 and NF-kB proteins, thereby decreased the expression of iNOS and the production of NO [35]. Moreover, in hippocampal neurons, daidzein was demonstrated to promote neurite outgrowth via ER-β, which in turn may increase the phosphorylation of PKCα and growth associated protein 43 (GAP-43) [22]. In contrast to these ER-dependent signaling systems, here our results revealed that the neuritogenic effects of daidzein in DRG neurons did not mediate through ER α/β or GPR-30 (the third kind of ER receptor), as pretreatment of DRG neurons with specific estrogen receptor antagonists, ICI 182780, tamoxifen, or G-15, did not block the daidzein-induced neuritogenesis.

Most importantly, our studies showed that daidzein treatment increased the phosphorylation of Src kinase and ERK1/ERK2 in cultured DRG neurons. Src kinase, which is activated by various molecules like NGF, laminin, artemin, and anti-Thy-1 antibody, has been shown to be an important signaling pathway involved in the process of DRG neurite outgrowth [27,36,37]. Downstream signaling of Src includes MEK/ERK and PI3K/Akt pathways, which can be activated by NGF to induce neurite extension and branching of DRG neurons [38]. While inhibition of Src kinase by PP2 and suppression of ERK1/2 by U0126 abolished the neuritogenic effect of daidzein, inhibition of Akt by LY294002 had no effect (unpublished observation). Thus, the current study did not support the role of PI3K/Akt pathway in daidzein-induced neuritogenesis. The increased phosphorylation of ERK and daidzein-induced neuritogenesis was blocked by the Src kinase inhibitor PP2, indicating that promotion of neurite outgrowth by daidzein required Src kinase and ERK. As phosphorylated ERK could activate CREB, Cdk5, GAP-43 and other neuritogenesis-related genes [39-41], it is possible that daidzein-induced neurite outgrowth is mediated by the Src-ERK pathway. Interestingly, PKCδ has been shown to be phosphorylated and activated by Src kinases in salivary and PC12 cells [42]. Consistent with this notion, we also found that daidzein increased the phosphorylation of PKCδ, and that inhibition of PKCδ by a selective PKCδ inhibitor rottlerin resulted in the suppression of neurite outgrowth, suggesting that PKCδ may also play a role in the signaling cascade induced by daidzein. Different PKC isozymes, including PKCδ, have been found to be activated by neurotrophic agents. For examples, in PC12 cells the activation of ERK by neuritogenic agents, fibroblast growth factor (FGF) and NGF, was dependent on the activation of PKCδ [43]. In diabetic rats, over-expression of PKCδ could ameliorate the retarded neurite outgrowth of DRG neurons [44]. Additional studies are therefore required to elucidate the mechanistic link between daidzein-induced activation of the Src-PKCδ-ERK pathway and the downstream signaling pathways that eventually lead to neuritogenesis.

As mentioned above, we have shown that daidzein-induced neuritogenesis in hippocampal and DRG neurons are mediated by ER-dependent [22] and ER-independent mechanisms, respectively. The precise reasons underlying this striking difference remain unclear. Intrinsic signaling pathways regulating neurite outgrowth could drastically vary among different types of neuronal cells [45,46]. For example, activation and inhibition of the small GTPase Rac1 promotes neurite outgrowths in hippocampal and DRG neurons, respectively [47,48]. Further experiments will be required to determine if the neuritogenic effect of daidzein is also differentially regulated in various regions of the nervous system.

In cultured rat hippocampal neurons, a low concentration of daidzein had neuroprotective action (at 3.9 μM), but it could not promote neuritogenesis or enhance neuronal survival [24]. Using relative affinity binding assay of cellular extracts, the affinity of daidzein for estrogen receptor was estimated to be several hundred times lower than estrogen [19]. Therefore, adequate amount of daidzein is required to achieve biological activities through the estrogen receptor pathways. Studies using daidzein at much higher concentrations (30 to 40 μM) in cultured hippocampal neurons indicate that daidzein can promote neurite extension and protect neurons from glutamate-induced cell death [22,24]. We demonstrated that daidzein at 30 μM increased neurite lengthening and branching for DRG neurons, which was in accordance with the results of previously published reports. In addition, we found 30 μM daidzein had a neuritogenic effect similar to that of NGF, indicating that daidzein had a robust neuritogenic property. Meanwhile, daidzein did not affect DRG neuronal survival at 30 μM. When daidzein was used at a concentration higher than 30 μM, the neuritogenesis was decreased. This finding is in agreement with the observed fact that both Src and ERK achieve the highest levels of phosphorylation at 30 μM daidzein.

Our finding that daidzein facilitates neurite outgrowth of DRG and hippocampal neurons has important implications for the potential facilitation of neural regeneration [49,50]. Preliminary study using daidzein for the treatment of optic nerve injury in rats have shown promising results [23]. Neurite outgrowth is a fundamental step in the establishment of neural connections during development and following injury. It would be interesting to see if daidzein could improve sensory and cognitive function in various animal disease models, e.g. injuries of peripheral nerves, brachial plexus, and spinal cord, as well as Alzheimer’s disease. On the other hands, the signaling mechanism of daidzein warrants further investigation. It has been shown that daidzein did not activate Src kinase via ERs, so other upstream regulators of Src could be the potential targets of daidzein. Many downstream effectors of ERK are associated with neurite outgrowth, and daidzein may preferentially activate some of those. Future research will be directed to tackle these questions.

## Conclusion

Daidzein enhances neurite outgrowth of cultured rat DRG neurons which is mediated by cooperative action of Src kinase, PKCδ, and MEK.

## Abbreviations

DIV: Day in vitro; DMSO: Dimethyl sulfoxide; DRG: Dorsal root ganglion; ER: Estrogen receptor; ERK: Extracellular signal-regulated kinase; GAP-43: Growth associated protein 43; GPR-30: G-protein coupled receptor 30; MEK: Mitogen-activated protein kinase/extracellular signal-regulated kinase kinase; MPP: 1-methyl-4-phenyl pyridium; MTT: 3-[4, 5-dimethylthiazol-2-yl]-2, 5-diphenyltetrazolium bromide; NF-L: Neurofilament light chain; NGF: Nerve growth factor; PBS: Phosphate buffered saline; PI3K: Phosphoinositide 3-kinase; PLC: Phospholipase C; PKC: Protein kinase C; PKCδ: Protein kinase C subtype delta; TBS: Tris-buffered saline.

## Competing interests

The authors declare that they have no competing interests.

## Authors’ contributions

SHY: designed and carried out experiments, analyzed results and manuscript writing. CCL, JPS: performed DRG cultures, immunostaining. YC: western blotting analysis. CJJ: designed experiments, analyzed results and manuscript revision. SMW: designed experiments, analyzed results and manuscript revision. All authors read and approved the final manuscript.
